# Particle bombardment and subcellular protein localization analysis in the aquatic plant *Egeria densa*

**DOI:** 10.7717/peerj.3779

**Published:** 2017-09-07

**Authors:** Yasuhide Osaki, Yutaka Kodama

**Affiliations:** Center for Bioscience Research and Education, Utsunomiya University, Utsunomiya, Tochigi, Japan

**Keywords:** Fluorescent protein, Aquatic plant, *Egeria densa*, Particle bombardment, Subcellular localization, Transient expression

## Abstract

Particle bombardment is a powerful and relatively easy method for transient expression of genes of interest in plant cells, especially those that are recalcitrant to other transformation methods. This method has facilitated numerous analyses of subcellular localization of fluorescent fusion protein constructs. Particle bombardment delivers genes to the first layer of plant tissue. In leaves of higher plants, epidermal cells are the first cell layer. Many studies have used the epidermal cell layer of onion bulb (*Allium cepa*) as the experimental tissue, because these cells are relatively large. However, onion epidermal cells lack developed plastids (i.e., chloroplasts), thereby precluding subcellular localization analysis of chloroplastic proteins. In this study, we developed a protocol for particle bombardment of the aquatic plant *Egeria densa*, and showed that it is a useful system for subcellular localization analysis of higher plant proteins. *E. densa* leaflets contain only two cell layers, and cells in the adaxial layer are sufficiently large for observation. The cells in both layers contain well-developed chloroplasts. We fused fluorescent proteins to conventional plant localization signals for the nucleus, cytosol, mitochondria, peroxisome, and chloroplast, and used particle bombardment to transiently express these fusion constructs in *E. densa* leaves. The plant subcellular localization signals functioned normally and displayed the expected distributions in transiently transformed *E. densa* cells, and even chloroplastic structures could be clearly visualized.

## Introduction

Subcellular localization analysis using fluorescent proteins (e.g., green fluorescent protein (GFP)) is a useful method to characterize genes and proteins of interest. In plant cells, transient gene expression is widely used to conduct subcellular localization analyses with fluorescent proteins. Methods of transient gene expression include infiltration of transgenic *Agrobacterium* harboring T-DNA and a gene of interest into leaf cells (Schöb, Kunz & Meins, 1997), polyethylene glycol (PEG)-mediated transformation of DNA into protoplasts (Krens et al., 1982), peptide-mediated DNA transfection (Lakshmanan et al., 2013), and bombardment of gold particles coated with DNA (particle bombardment) (Klein et al., 1987). Particle bombardment is widely used and efficiently transforms many plant species that are recalcitrant to other methods of transformation. Particle bombardment has been extensively used for transient transformation of the epidermal cell layer of onion bulb (*Allium cepa*) followed by subcellular localization analysis of plant proteins fused with fluorescent proteins (e.g., Kodama, 2011). However, onion epidermal cells contain only proplastids (undifferentiated plastids), and no chloroplasts (differentiated plastids) that emit autofluorescence, and the proplastids are morphologically similar to other organelles such as mitochondria and peroxisomes. Therefore, in onion cells, it is challenging to determine whether a protein of interest localizes to proplastids or to some other organelle (e.g., mitochondria and peroxisomes) that is similar to proplastids.

**Figure 1:**
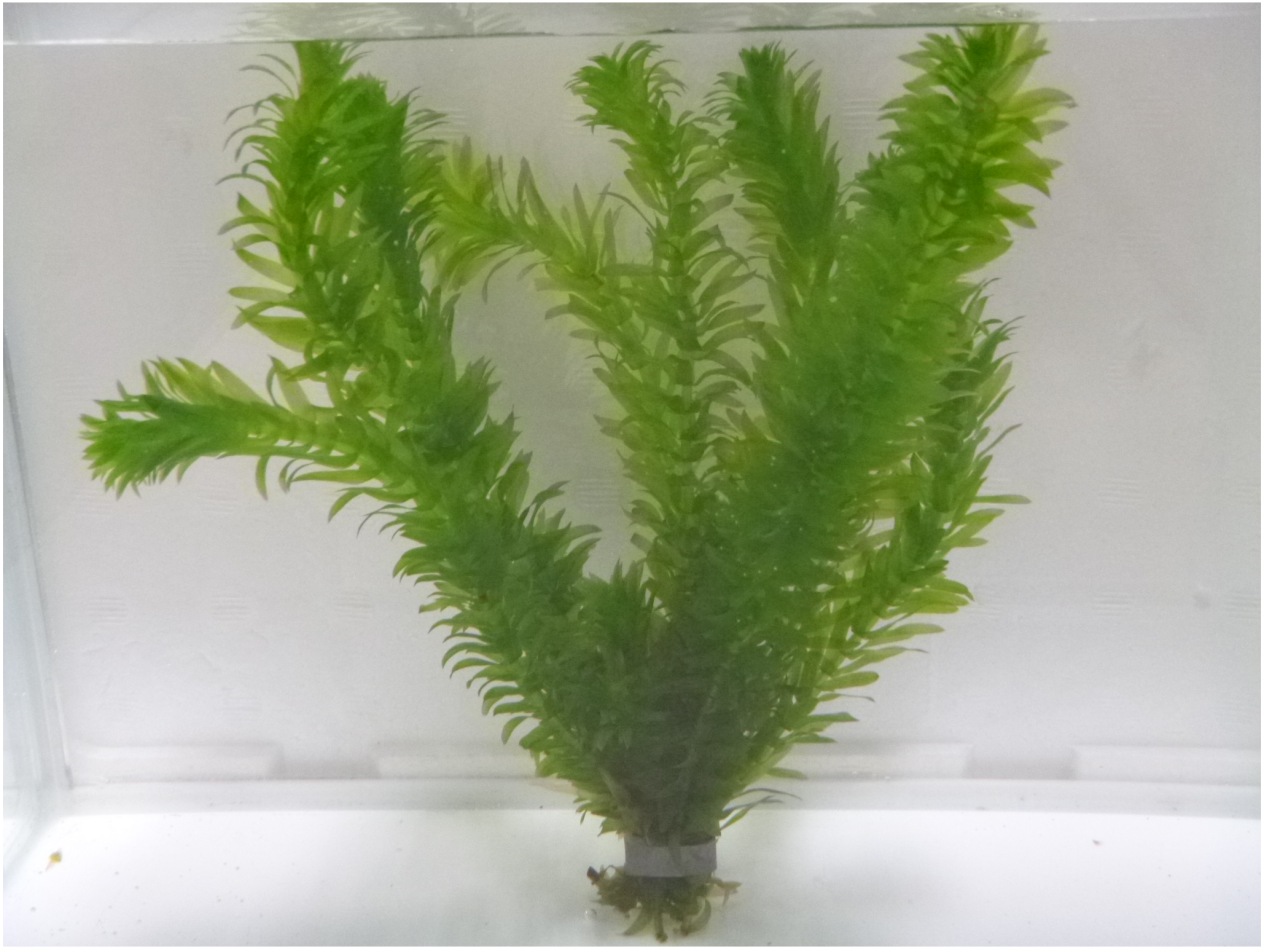
The aquatic monocot plant *Egeria densa.* Photograph of *Egeria densa* in fresh water.

*Egeria densa* is an aquatic monocot that lives in fresh water (Fig. 1) and belongs to the Hydrocharitaceae family. *E. densa* is commercially available as an ornamental plant called Anacharis (tradename) for aquariums, and is readily available where aquarium supplies are sold. *E. densa* leaves contain two single-cell layers. Chloroplasts are well developed in the cells of both layers. To determine if *E. densa* leaf cells could be used to analyze the subcellular localization of plant proteins, including chloroplast proteins, we tested whether *E. densa* could be transiently transformed for ectopic gene expression using particle bombardment.

## Materials and Methods

### Plant materials

*E. densa* plants were purchased from the aquarium store Kanseki Co., Ltd. (Utsunomiya, Japan), and cultured in fresh water under continuous dim white light (approximately 13 µmol photons m^−2^ s^−1^) at 22 °C before use for particle bombardment. Onion bulbs (*Allium cepa*) were purchased from the supermarket (Utsunomiya, Japan). The onion was kept at room temperature before being used for particle bombardment.

**Table 1:** Primers used in this study.

No.	Sequence in the 5′-to-3′ direction
1	GGGGACAAGTTTGTACAAAAAAGCAGGCTCCATGGTGAGCAAGGGCGAG
2	GGGGACCACTTTGTACAAGAAAGCTGGGTCTCACTTGTACAGCTCGTCC
3	GGGGACCACTTTGTACAAGAAAGCTGGGTCTCACACCTTGCGCTTCTTCTTAGGAGGCTTGTACAGCTCG
4	GGGGACAAGTTTGTACAAAAAAGCAGGCTCCATGATGATGATGAATCT
5	GCCCTTGCTCACCATCTTTGAAAGAAATGA
6	ATGGTGAGCAAGGGCGAGGAGCTGTTCACC
7	GGGGACCACTTTGTACAAGAAAGCTGGGTCTCACAACTTGGACTTGTACAGCTCGTCC
8	GGGGACAAGTTTGTACAAAAAAGCAGGCTTCATGGCTTCCTCTATGCTC
9	CCTTAGACACCATGGAATCGGTAAGGTCAGGAAG
10	CCTTACCGATTCCATGGTGTCTAAGGGCGAAG
11	GGGGACCACTTTGTACAAGAAAGCTGGGTCTCAATTAAGTTTGTGCCCC
12	GGGGACAAGTTTGTACAAAAAAGCAGGCTCCATGGGAAAAACTTCGGG
13	CTCGCCCTTGCTCACCATGTCATCGGGGTCTTTG
14	ACCAAAGACCCCGATGACATGGTGAGCAAGGGCG
15	GGGGACCACTTTGTACAAGAAAGCTGGGTCCTTGTACAGCTCGTCCATG
16	GGGGACAAGTTTGTACAAAAAAGCAGGCTTCATGGTGAGCAAGGGCGAGGAGGA
17	GGGGACCACTTTGTACAAGAAAGCTGGGTCCTTGTACAGCTCGTCCATG

### Plasmid construction

The primers (No. 1–17) used in this study are listed in Table 1. All PCR experiments were performed with the PrimeSTAR® MAX DNA Polymerase (Takara Bio, Kyoto, Japan) and GeneAmp® PCR System 9700 (Perkin-Elmer, Waltham, MA, USA).

**Figure 2:**
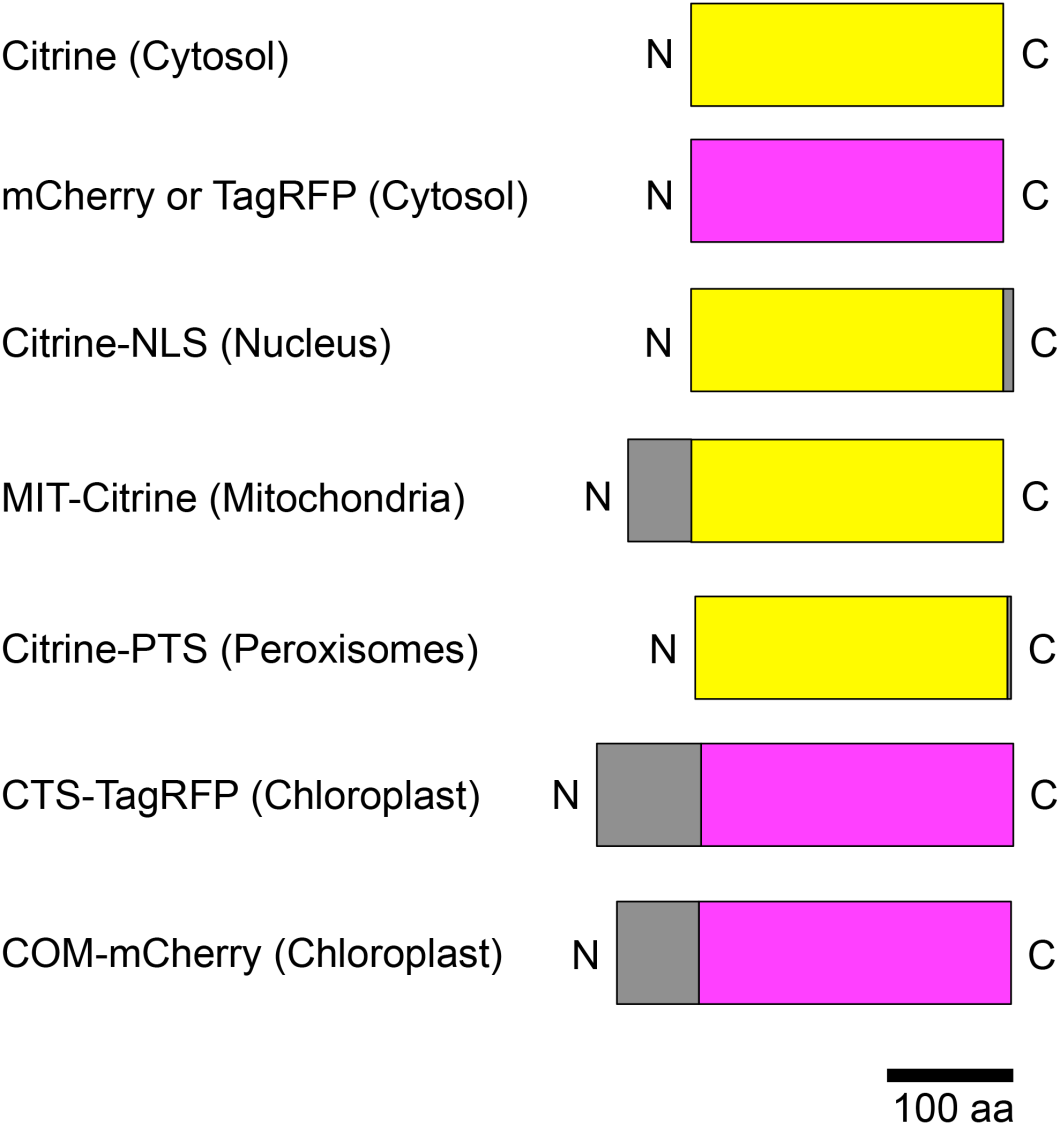
Schematic illustration of fusion protein constructs. Citrine was used as the yellow fluorescent protein (yellow boxes); mCherry and TagRFP were used as the red fluorescent proteins (magenta boxes). Subcellular localization signals are shown as gray boxes and defined in parentheses. NLS, nuclear localization signal; MIT, mitochondrial localization signal; PTS, peroxisomal targeting signal; CTS, chloroplast/plastid transit signal; COM, chloroplast outer envelope membrane localization signal; N, N-terminus; C, C-terminus.

To construct the pGWT35S-Citrine plasmid expressing cytosolic fluorescent protein, *Citrine* was PCR-amplified using pMpGWB106 (Ishizaki et al., 2015) as template with primers No. 1 and No. 2. To construct the pGWT35S-Citrine-NLS plasmid expressing a Citrine-NLS fusion protein, *Citrine-NLS* was PCR-amplified using primers No. 1 and No. 3. To construct the pGWT35S-TIM21(1–50)-Citrine plasmid expressing a mitochondrial targeting signal fusion, a cDNA fragment for the *Arabidopsis translocase of inner mitochondrial membrane 21* (*TIM21* (1–50 aa)) homolog was PCR-amplified using pMpGWB106-TIM21(1–50) as template with primers No. 4 and No. 5, and *Citrine* was PCR-amplified using pMpGWB106-TIM21(1–50) (Ogasawara et al., 2013) as template with primers No. 2 and No. 6. Then, the *TIM21* and *Citrine* fragments were mixed and joined by PCR amplification using primers No. 2 and No. 4 to produce *TIM21* (1–50)-*Citrine*. To construct the pGWT35S-Citrine-PTS plasmid expressing fluorescent protein in the peroxisome, *Citrine-PTS* was PCR-amplified using pMpGWB106 (Ishizaki et al., 2015) as template with primers No. 1 and No. 7. To construct the pGWT35S-RBCS1a(1–79)-TagRFP plasmid expressing fluorescent protein in the chloroplast (plastid), the cDNA fragment for *Arabidopsis ribulose bisphosphate carboxylase small chain 1A* (1–79 aa) was PCR-amplified using a cDNA library, which was prepared from rosette leaves of *Arabidopsis thaliana*, as template with primers No. 8 and No. 9, and *TagRFP* was PCR-amplified using TagRFP cDNA, which was artificially synthesized (Operon), as template with primers No. 10 and No. 11. The resulting two fragments were joined by PCR amplification using primers No. 8 and No. 11 to produce *RBCS1a* (1–79)*-TagRFP*. To construct the pGWT35S-OEP7-mCherry plasmid expressing fluorescent protein at the chloroplastic (plastid) outer envelope membrane, the cDNA fragment for *Arabidopsis outer envelope membrane protein 7* was PCR-amplified using pDONR207-OEP7 (Tanaka et al., in press) as template with primers No. 12 and No. 13, and mCherry was PCR-amplified using pDONR207-mCherry as template with primers No. 14 and No. 15. Note that pDONR207-mCherry was previously prepared by PCR amplification using pmCherry (Cat. No. 632522; Clontech, Mountain View, CA, USA) as template and primers No. 16 and No. 17, and Gateway cloning. The resulting two fragments were joined by PCR amplification using primers No. 12 and No. 15 to produce *OEP7-mCherry*. These fusion constructs targeted to the cytosol (*Citrine*), nucleus (*Citrine-NLS*), mitochondrion (*TIM21* (1–50)-*Citrine*), peroxisome (*Citrine-PTS*), chloroplast stroma (*RBCS1a* (1–79)*-TagRFP*), and chloroplast outer envelope membrane (*OEP7-mCherry*) were cloned into pDONR207, and then transferred into pGWT35S (Y Fujii, A Yoshimura & Y Kodama, 2017, unpublished data) via Gateway cloning technology (Invitrogen) to produce pGWT35S-Citrine, pGWT35S-Citrine-NLS, pGWT35S-TIM21(1–50)-Citrine (renamed pGWT35S-MIT-Citrine), pGWT35S-Citrine-PTS, pGWT35S-RBCS1a(1–79)-TagRFP (renamed pGWT35S-CTS-TagRFP), and pGWT35S-OEP7-mCherry (renamed pGWT35S-COM-mCherry), respectively (Fig. 2). Although cloning of *Citrine-NLS* and *Citrine-PTS* into pDONR207 was reported previously (Ogasawara et al., 2013), the primers used to generate these plasmids in the present study are listed in Table 1.

**Figure 3:**
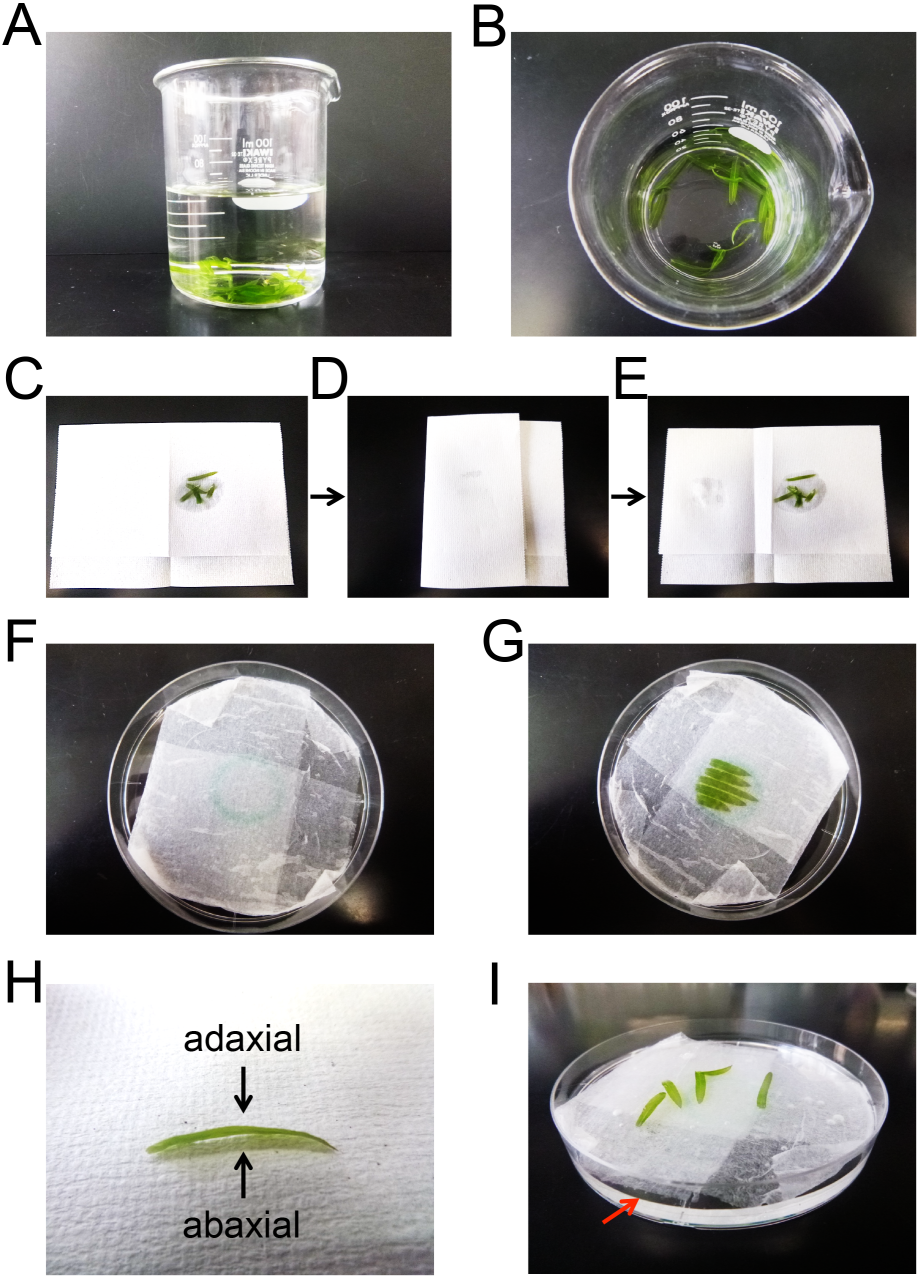
Preparation of *E. densa* leaflets for particle bombardment. (A and B) Representative snapshot images of detached leaflets in distilled water. (C–E) Representative snapshot images of leaflets that had been blotted with dry paper towel to remove excess water from the leaflet surface. (F) A representative snapshot image of wet Kimwipes in a 9 cm Petri dish. A circle was drawn at center of the dish. (G) A representative snapshot image of the leaflets placed on wet Kimwipes in a 9 cm Petri dish. (H) A representative snapshot image of a detached leaflet. Arrows indicate the adaxial and abaxial surfaces. (I) A representative snapshot image of the leaflets in water in a 9 cm Petri dish. The red arrow indicates the surface of the water.

### Particle bombardment

One microgram of prepared plasmid (pGWT35S-Citrine, pGWT35S-Citrine-NLS, pGWT35S-MIT-Citrine, pGWT35S-Citrine-PTS, pGWT35S-CTS-TagRFP, or pGWT35S-COM-mCherry) and 1 µg of control plasmid (encoding cytosolic mCherry or Citrine) were mixed in 10 µL of sterile water, and coated onto 0.6 mg of gold particles (1 µm diameter). Onion bulb scale leaves and *E. densa* leaflets were prepared for bombardment. The onion bulb scale leaves were cut into strips of approximately 5 cm × 3 cm, and a strip of scale leaf was placed on a stack of wet Kimwipes in a 9 cm Petri dish. *E. densa* leaflets were detached and kept in distilled water until bombarded with DNA-coated gold particles (Figs. 3A and 3B). To increase the transformation efficiency, the leaflets were blotted with dry paper towels (or dry Kimwipes) to remove excess water from the leaflet surface before bombardment (Figs. 3C–3E). Five *E. densa* leaflets were placed on a stack of wet Kimwipes in the center of a 9 cm Petri dish (Figs. 3F and 3G). Either the adaxial or abaxial surfaces, which could be distinguished based on the curvature of the leaflets (Fig. 3H), were facing upwards. Then, the strips of onion scale leaves and the *E. densa* leaflets were subjected to particle bombardment using the biolistic PDS1000/He Particle Delivery System (Bio-Rad) (Kodama, 2011). Bombardment was performed with a 650 psi rupture disc and 590 MPa pressure. In preliminary experiments, we tested three types of rupture discs (450 psi, 650 psi, and 1,100 psi), and found that sufficient Citrine expression was achieved with 650 psi and 1,100 psi rupture discs. To avoid cellular damage during bombardment, we selected 650 psi rupture discs for future experiments. Citrine-NLS, MIT-Citrine, or Citrine-PTS was co-expressed with cytosolic mCherry, and CTS-TagRFP or COM-mCherry was co-expressed with cytosolic Citrine. Cytosolic mCherry and Citrine were used to visualize cell shape and served as expression controls.

**Figure 4:**
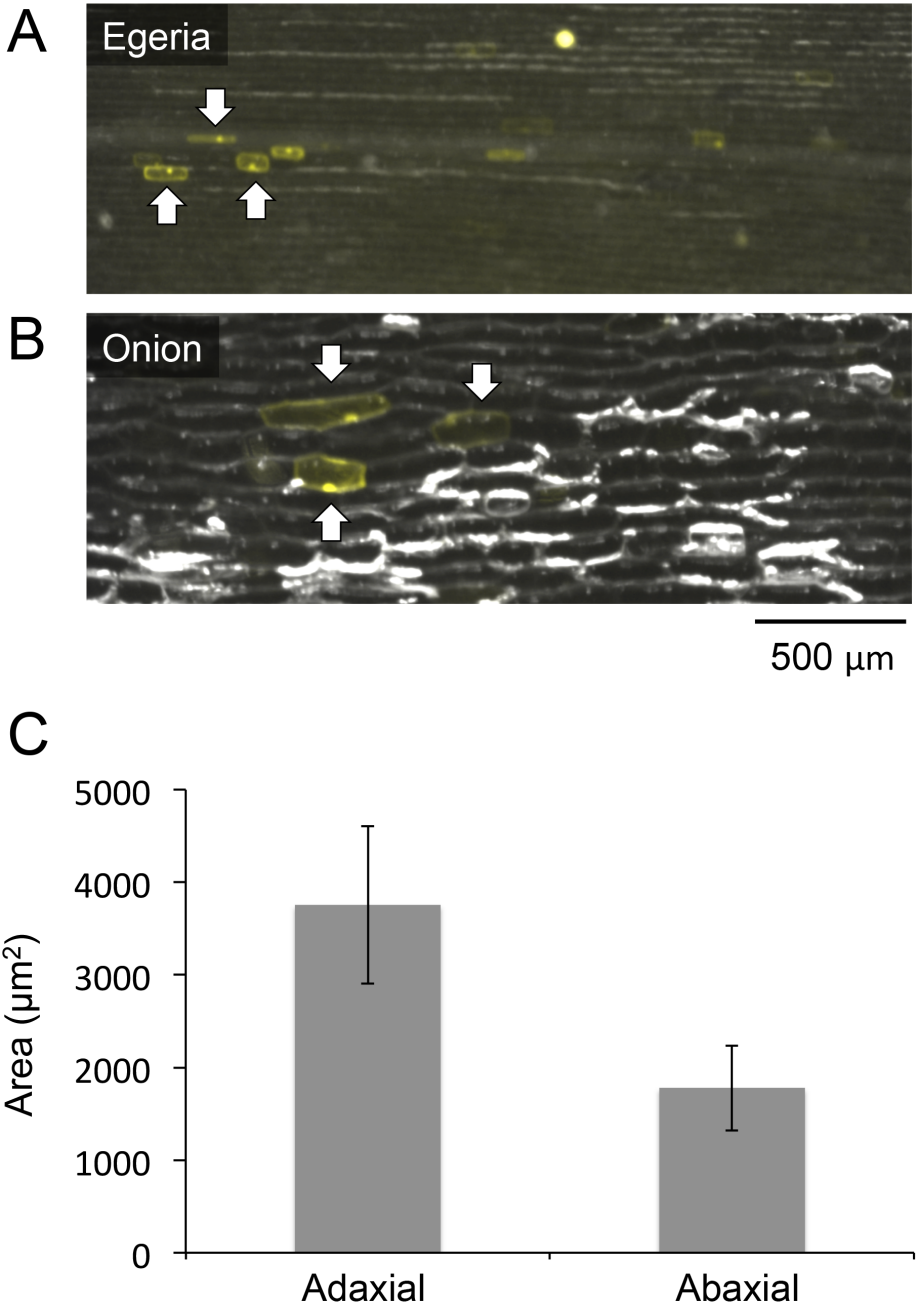
Comparisons of cell sizes in *Egeria* adaxial cells and onion epidermal cells. Observations of *Egeria* adaxial cells (A) and onion epidermal cells (B) under a stereo fluorescence microscope. Arrows indicate cells transiently expressing Citrine fluorescent protein. (C) Comparisons between adaxial and abaxial cells in *Egeria densa* leaflets. Each cell area (*n* = 20) was measured using ImageJ; average cell sizes and standard deviations (shown as error bars) were obtained.

### Microscopy analysis

After bombardment, distilled water was poured into the Petri dishes to keep the samples moist (Fig. 3I). The samples in the Petri dishes were incubated in darkness at 22 °C for 24 h, and cells were observed using a confocal laser scanning microscope (SP8X; Leica Microsystems) and the time-gating method (Kodama, 2016). Onion epidermal cells were used as controls. Citrine fluorescence was observed with 513 nm excitation and 517–548 nm emission. mCherry fluorescence was observed with 587 nm excitation and 596–628 nm emission. TagRFP fluorescence was observed with 555 nm excitation and 607–640 nm emission. Chlorophyll autofluorescence was observed with excitation at 513 and 587 nm (or 555 nm) and 639–714 nm (or 701–776 nm) emission. Propidium iodide (PI) (Invitrogen, Carlsbad, CA, USA) and MitoTracker Red CMXRos (Molecular Probes, Eugene, OR, USA) were treated with bombarded cells before observation. The PI fluorescence was observed with 547 nm excitation and 633–652 nm emission. The MitoTracker fluorescence was observed with 579 nm excitation and 631–652 nm emission. For imaging of Citrine fluorescence in cells stained with PI or MitoTracker, 513 nm excitation and 517–546 nm emission were used.

## Results and Discussion

We analyzed the relative sizes of *E. densa* adaxial leaf cells and onion cells using a stereo fluorescence microscope (M205FA; Leica Microsystems). Although *Egeria* cells were smaller than onion cells, they were large enough (length: ∼150 μm, width: ∼50 μm) for microscopy observation (Figs. 4A and 4B). Particle bombardment was used to transiently express Citrine fluorescent protein (Fig. 2) in *Egeria* cells, and the cells clearly emitted yellow fluorescence even under low magnification with a stereo fluorescence microscope (Fig. 4A). Then, we compared the sizes of *E. densa* adaxial and abaxial cells, and found that the adaxial cells were 2-fold larger than the abaxial cells (Fig. 4C and raw data in Data S1). This indicates that cells in the adaxial cell layer are appropriate for protein subcellular localization analysis.

**Figure 5:**
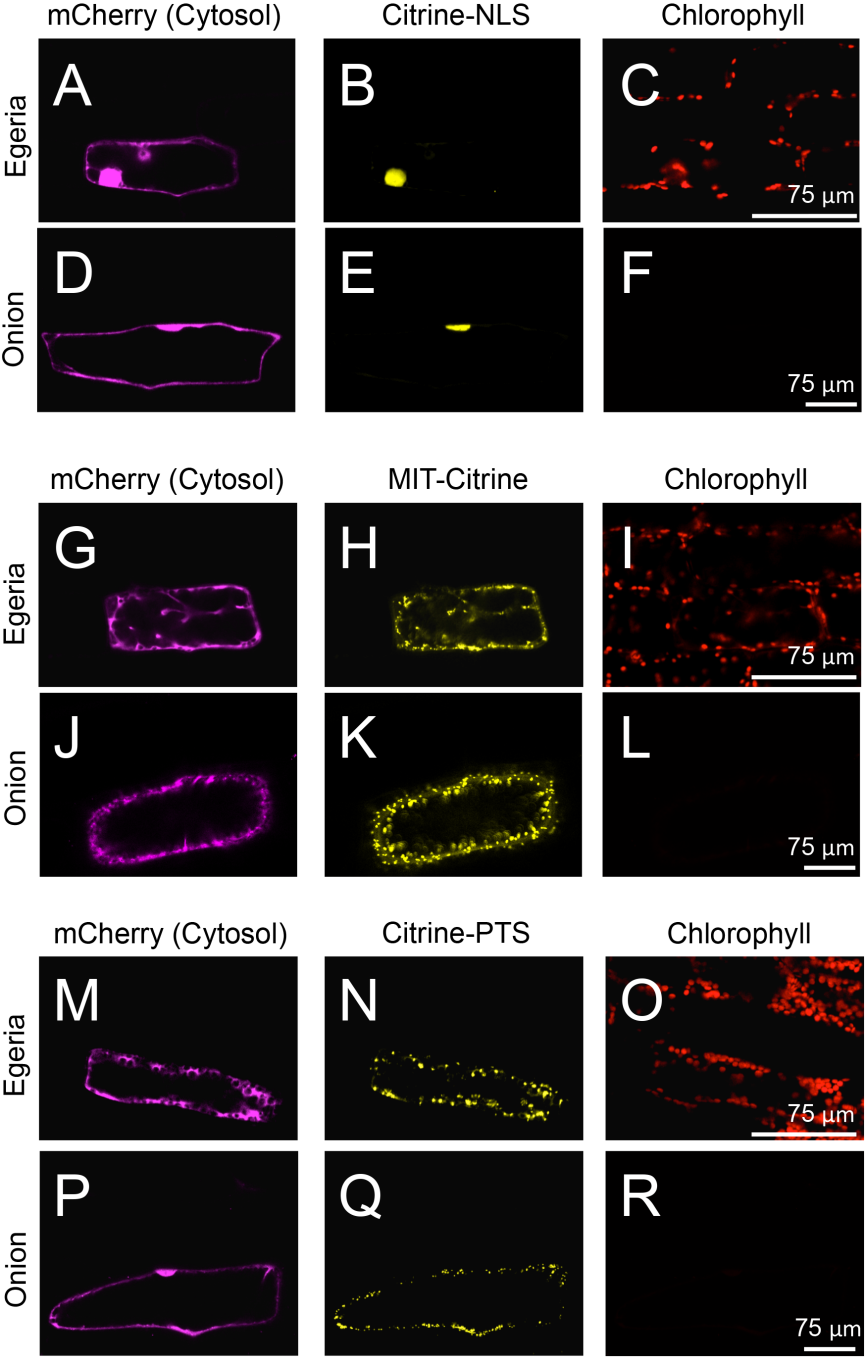
Transient expression of Citrine-NLS, MIT-Citrine, or Citrine-PTS in onion and *Egeria* cells. Citrine-NLS (A–F), MIT-Citrine (G–L), or Citrine-PTS (M–R) was transiently expressed in *Egeria* and onion cells via particle bombardment. The cells were observed using confocal microscopy.

To test the subcellular localization of conventional land plant signal peptides in *Egeria* cells, we constructed fluorescent protein fused with various signal peptides (Fig. 2). Cytosolic fluorescent proteins (Citrine, TagRFP, and mCherry) were fused with nuclear localization signal (NLS) (Kalderon et al., 1984; Ogasawara et al., 2013), mitochondrial targeting signal (MTS) (Hamasaki et al., 2012; Ogasawara et al., 2013), peroxisomal targeting signal (PTS) (Gould, Keller & Subramani, 1987; Gould et al., 1989; Ogasawara et al., 2013), chloroplast/plastid transit signal (CTS) (Lee et al., 2009), and chloroplast outer envelope membrane localization signal (COM) (Lee et al., 2001; Tanaka et al., in press). The fluorescent fusion proteins were transiently transformed in the adaxial cells using particle bombardment and observed using the time-gating method (Kimura & Kodama, 2016; Kodama, 2016), with the gate time set at 0.5–12.0 ns to block chlorophyll autofluorescence. The observed intracellular distributions of fluorescent fusion proteins in the transformed *Egeria* cells were consistent with the expected distributions for signal peptides targeting to the nucleus, mitochondria, and peroxisomes (Figs. 5A–5R).

**Figure 6:**
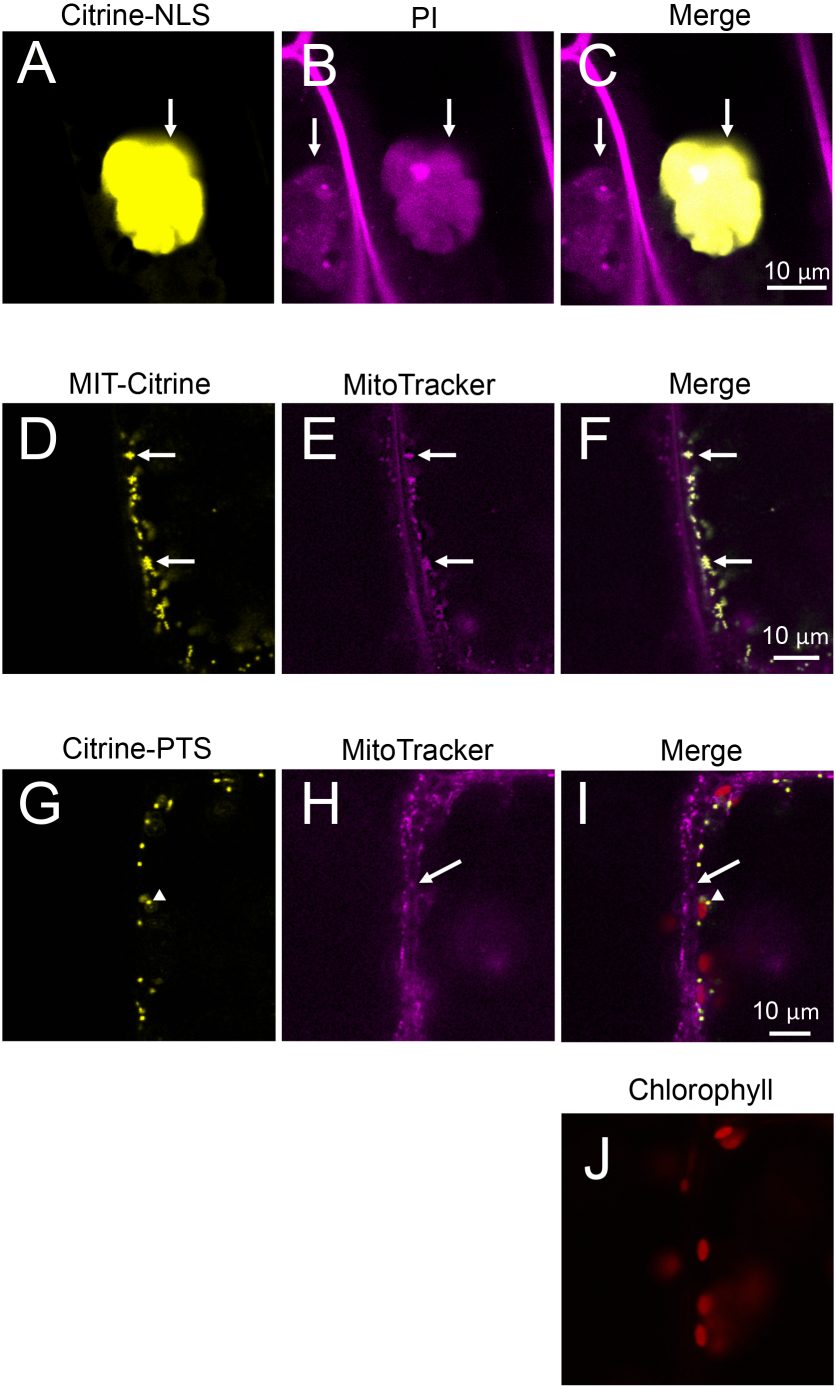
Characterization of organelles visualized by Citrine-NLS, MIT-Citrine, and Citrine-PTS. (A–C) Propidium iodide (PI)-stained cells expressing Citrine-NLS. Before observation, the bombarded cells were treated with PI solution (0.5 mg/mL) (Invitrogen, Carlsbad, CA, USA) for 8 h. Arrows indicate nuclei. (D–F) MitoTracker-stained cells expressing MIT-Citrine. Before observation, the bombarded cells were treated with MitoTracker Red CMXRos (200 nM) (Molecular Probes, Eugene, OR, USA) for 2 min. Arrows indicate mitochondria. (G–J) Comparison of the organelles expressing Citrine-PTS with mitochondria, and interaction of the organelles with chloroplasts. Before observation, the bombarded cells were treated with MitoTracker Red CMXRos (200 nM) (Molecular Probes, Eugene, OR, USA) for 2 min. Arrows and arrowheads indicate mitochondria and peroxisomes, respectively.

Nuclear localization of Citrine-NLS and mitochondrial localization of MIT-Citrine were confirmed by staining with propidium iodide (PI) and MitoTracker, respectively (Figs. 6A–6F). However, no chemical stain is available for peroxisomes. It was reported that peroxisomes interact with chloroplasts in *Marchantia polymorpha* and *Arabidopsis thaliana* (Ogasawara et al., 2013; Oikawa et al., 2015). Similar to the peroxisomes of *M. polymorpha* and *A. thaliana*, Citrine-PTS-localized organelles interacted with *E. densa* chloroplasts (Figs. 6G–6J). We also confirmed that the Citrine-PTS-localized organelles were not mitochondria (Figs. 6G–6J). Thus, we conclude that the Citrine-PTS-localized organelles in *E. densa* are peroxisomes.

**Figure 7:**
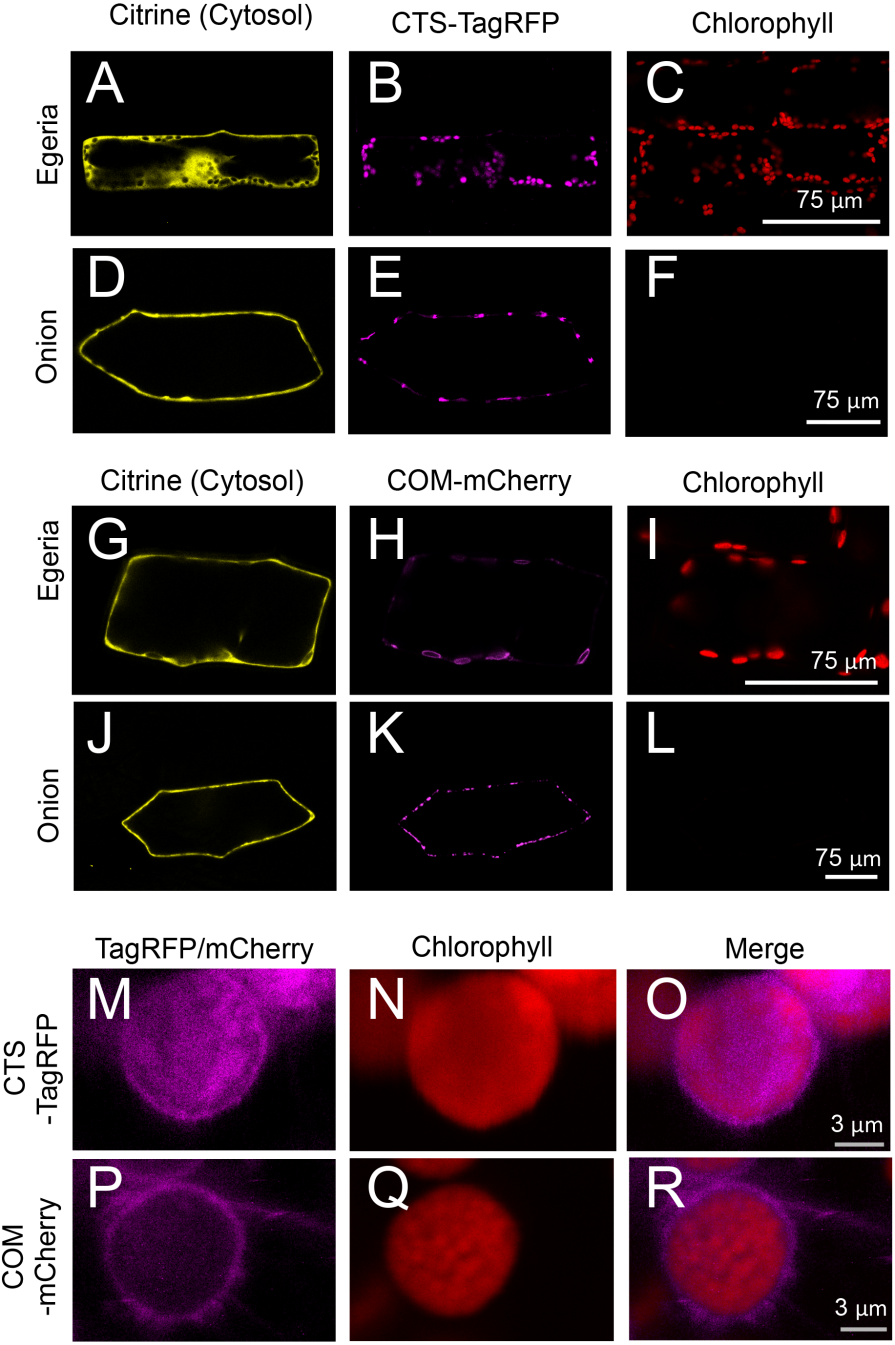
Transient expression of CTS-TagRFP or COM-mCherry in onion and *Egeria* cells. CTS-TagRFP (A–F) or COM-mCherry (G–L) was transiently expressed in *Egeria* and onion cells via particle bombardment. (M–R) Enlarged image of chloroplasts in cells expressing CTS-TagRFP or COM-mCherry. Cells were observed using confocal microscopy.

The subcellular distribution of fluorescent fusion proteins targeted to chloroplasts (CTS-TagRFP and COM-mCherry) also clearly co-localized with chlorophyll autofluorescence (Figs. 7A–7L). The dimensions of plastids (Figs. 7E and 7K), mitochondria (Fig. 5K), and peroxisomes (Fig. 5Q) were essentially equivalent in onion epidermal cells, and we could not detect chlorophyll autofluorescence in these cells (Figs. 7F and 7L). Therefore, we could not identify the subcellular localization of chloroplast proteins in onion cells. By contrast, chloroplast proteins were clearly identified in *Egeria* cells due to chloroplast autofluorescence (Figs. 7B, 7C, 7H and 7I). The size and shape of *E. densa* chloroplasts are normally uniform and round, respectively (e.g., Figs. 7B and 7C). However, we also observed large and oval chloroplasts in some cells (e.g., Figs. 7H and 7I). Although the causes for the size and shape changes remain to be determined, these changes would not interfere with the subcellular localization analysis. The chloroplast stroma and outer envelope membrane were clearly differentiated in enlarged images of chloroplasts in transiently transformed *Egeria* cells expressing CTS-TagRFP or COM-mCherry fusion proteins, respectively (Figs. 7M–7R). These results indicate that *E. densa* is a suitable plant species for transient transformation via particle bombardment and subsequent analysis of the subcellular localization of plant chloroplast/plastid proteins.

*E. densa* is a popular ornamental plant widely used in aquariums, and, like onion bulbs, is relatively inexpensive. Because all of the experimental procedures except the preparation of plant materials were the same in the *E. densa* and onion systems, experimental cost and time are comparable between the *E. densa* and onion systems.

Although particle bombardment can readily be used to transiently introduce genes into cells, it is not suitable for transformation of mesophyll cells, because the mesophyll cells, which contain differentiated chloroplasts, are located in the second cell layer (i.e., beneath the first epidermal cell layer) of the leaf in many land plants. To date, subcellular localization analysis of chloroplast proteins with chlorophyll autofluorescence was performed using PEG- and *Agrobacterium*-mediated transient expression systems and mesophyll cells of land plants such as *A. thaliana*. However, for the PEG- or *Agrobacterium*-mediated transient expression systems (Krens et al., 1982; Schöb, Kunz & Meins Jr, 1997), preparations of mesophyll protoplast cells (material) or transgenic *Agrobacterium* (carrier) are needed, respectively. In contrast to those systems, our *Egeria* bombardment system can readily be used to observe the localization of chloroplast proteins fused with fluorescent protein. In our system, the procedure for preparing the material and carrier is simple. Thus, we believe that the *Egeria* bombardment system will greatly facilitate studies of chloroplast proteins.

The *E. densa* system is useful for analyzing the intrachloroplast localizations of proteins and protein complexes. In chloroplasts, several compartments are differentiated, including the thylakoid membrane, stroma, and grana. Recently, we developed a time-gating method to block chlorophyll autofluorescence for clearer fluorescence imaging in plant cells (Kodama, 2016). Using the time-gating method to examine fluorescently tagged proteins is a robust method to observe chloroplast proteins fused with fluorescent protein in the chloroplast. Given that fluorescent proteins are readily observed in chloroplasts, protein complexes can also be observed using bimolecular fluorescence complementation (BiFC) (Kodama & Hu, 2012) in chloroplasts. Thus, the *E. densa* system presented here is an emerging model system for studies of chloroplast proteins and protein complexes.

## Conclusions

In this study, we constructed fluorescent fusion proteins containing specific subcellular targeting sequences of higher plants, and introduced these constructs into leaflets of the aquatic monocot *E. densa* using particle bombardment. The transiently transformed leaflets successfully expressed the plant signal peptides in the expected subcellular compartments. Unlike onion epidermal cells, *Egeria* cells contain chloroplasts. Chloroplast autofluorescence provides an internal marker that can be used to validate the subcellular localization of chloroplastic proteins. These combined results indicate that *E. densa* leaflets are a superior model system for rapid analysis of the subcellular localization of plant proteins.

##  Supplemental Information

10.7717/peerj.3779/supp-1 Data S1 Raw data for Fig. 3B

## Additional Information and Declarations

### Data Availability

The following information was supplied regarding data availability: The raw data has been supplied as Data S1.
